# Chikungunya Virus, Metabolism, and Circadian Rhythmicity Interplay in Phagocytic Cells

**DOI:** 10.3390/metabo13111143

**Published:** 2023-11-11

**Authors:** Linamary Alvarez-García, F. Javier Sánchez-García, Mauricio Vázquez-Pichardo, M. Maximina Moreno-Altamirano

**Affiliations:** 1 Laboratorio de Inmunorregulación, Departamento de Inmunología, Escuela Nacional de Ciencias Biológicas del IPN, Prolongación de Carpio y Plan de Ayala s/n, Col. Casco de Santo Tomás, Mexico City 11340, Mexico; linamaryalvarezgarcia@gmail.com (L.A.-G.); fjsanchez@ipn.mx (F.J.S.-G.); mauricio.vazquez@salud.gob.mx (M.V.-P.); 2 Laboratorio de Arbovirus, Departamento de Virología, Instituto de Diagnóstico y Referencia Epidemiológicos (InDRE), Secretaría de Salud, Francisco de P. Miranda 177, Col. Lomas de Plateros, Mexico City 01480, Mexico

**Keywords:** chikungunya virus, macrophages, metabolic reprogramming, circadian immunovirometabolism, tricarboxylic acid cycle

## Abstract

Chikungunya virus (CHIKV) is transmitted to humans by mosquitoes of the genus *Aedes*, causing the chikungunya fever disease, associated with inflammation and severe articular incapacitating pain. There has been a worldwide reemergence of chikungunya and the number of cases increased to 271,006 in 2022 in the Americas alone. The replication of CHIKV takes place in several cell types, including phagocytic cells. Monocytes and macrophages are susceptible to infection by CHIKV; at the same time, they provide protection as components of the innate immune system. However, in host–pathogen interactions, CHIKV might have the ability to alter the function of immune cells, partly by rewiring the tricarboxylic acid cycle. Some viral evasion mechanisms depend on the metabolic reprogramming of immune cells, and the cell metabolism is intertwined with circadian rhythmicity; thus, a circadian immunovirometabolism axis may influence viral pathogenicity. Therefore, analyzing the interplay between viral infection, circadian rhythmicity, and cellular metabolic reprogramming in human macrophages could shed some light on the new field of immunovirometabolism and eventually contribute to the development of novel drugs and therapeutic approaches based on circadian rhythmicity and metabolic reprogramming.

## 1. Introduction

Chikungunya fever is a disease caused by the chikungunya virus (CHIKV), transmitted to humans by infected mosquitoes, mainly *Aedes aegypti* and *Aedes albopictus*. This virus was detected for the first time in southern Tanzania, back in 1952. Currently, CHIKV circulates in Asia, Africa, and Europe; in 2013, the presence of this virus was registered in several regions of the Americas, with some fluctuation in the number of cases per year. In 2022, 271,006 cases of chikungunya were reported in the Americas, along with 94 related deaths [1].

Symptoms of infection begin 4 to 8 days after the mosquito bite; the most common is fever, usually accompanied by joint pain and other symptoms such as muscle pain, headache, nausea, fatigue, and rash. Severe joint pain often lasts a few days, but may persist for months or even years [1]. The World Health Organization (WHO) defines four clinical forms of chikungunya disease: chronic, acute, severe acute, and atypical [2]. Severe complications are rare, but in the elderly, the disease may lead to permanent immobility and even death [1]. 

The re-emergence of CHIKV and the magnitude of outbreaks associated with it highlight the need for more research in the field. In 2018, CHIKV was added to the WHO list of research and development priorities [1]. It is worth mentioning that vaccines for this virus are not yet commercially available.

The concepts of and experimental approaches to immunometabolism, circadian rhythmicity, and chronotherapy are only recently being incorporated into the study of CHIKV infection. Studies on the metabolic reprogramming caused by viral infections such as CHIKV might contribute to understanding the mechanisms of infection and evasion of the immune response. 

Host defense mechanisms can detect virus infection and then activate antiviral programs; in addition, viruses modify the metabolic profile of infected cells [3]. Thus, viruses and their host cells seem to struggle for the control of cellular metabolism in order to promote or inhibit viral replication, respectively.

Recent research has demonstrated a connection between the immune system, circadian rhythms, and metabolism in the context of viral infections, and there is evidence that innate immunity is strongly influenced by the time of day [4,5,6,7,8,9]. 

This circadian/metabolic axis can influence the viral replicative cycle and could be a key factor in driving the rhythmicity of the immune function; thus, this is also a topic that should be considered for immunometabolism studies (circadian immunovirometabolism).

## 2. Chikungunya Virus

CHIKV belongs to the *Togaviridae* family and the Alphavirus genus. It is a virus of 60-70 nm in diameter, with a lipid membrane and an icosahedral capsid. It has a positive single-stranded RNA genome of approximately 11.8 kb with a 5′-methylguanylate cap and a 3′-polyadenylate tail encoding for four nonstructural proteins (nsP1 to nsP4), three structural proteins (capsid [C], envelope [E1 and E2]), and two small peptides (E3 and 6 K) in two open reading frames [10,11].

Like other mosquito-borne arboviruses, CHIKV enters the skin during the ingestion of blood by an infected female mosquito. Initial virus replication in dermis-residing cells allows the viruses to enter the bloodstream and spread through the lymphatic fluid, reaching the lymph nodes via infected migratory cells [12,13]. In this way, viruses have access to various parts of the body accompanied by a marked infiltration of immune cells [14]. 

CHIKV infects a wide variety of cell types in both their mammalian host and the mosquito. This broad cell tropism might be due to the ability of CHIKV to bind to a diversity of entry receptors.

Different studies demonstrate that several cell lines are also susceptible to in vitro infection, paving the way for future research on CHIKV-related pathogenic mechanisms. 

Studies for mosquito cells, CCL-125 from *Aedes aegypti* and C6/36 from *Aedes albopictus*, and on mammalian cell lines such as HepG2 (human hepatocarcinoma), HeLa (human cervical epithelium), HEK293T/17 (human embryonic kidney), CHME-5 (human embryonic fetal microglia), MRC-5 (human pulmonary fibroblast), Vero (African green monkey kidney), and BHK-21, J774, RAW 264.7 (mouse macrophages), have been evaluated for CHIKV infection [15,16,17,18].

The entry of CHIKV into host cells is more likely via clathrin-mediated endocytosis, upon binding to a membrane receptor [19,20]. This original hypothesis was reinforced by the discovery and in vivo validation of MXRA8 as a CHIKV receptor in human cells [21]. Nevertheless, incomplete in vivo suppression of CHIKV replication in mice by inhibition of the MXRA8 receptor [21,22] suggests the presence of other receptors or binding factors, such as heparin and heparan sulfate, prohibitin, T cell/transmembrane immunoglobulin and mucin domain protein 1 (TIM1), dendritic cell intercellular adhesion molecule 3 (DC-SIGN), and basigin or EMMPRIN (extracellular matrix metalloproteinase inducer) (CD147) [23,24,25,26]. No ortholog of MXRA8 has been found in mosquito cells, which highlights the apparent diversity of cellular entry factors for CHIKV [27]. Although heat shock cognate 71 kDa protein (HSC70) and the ATP5A1 subunit of adenosine triphosphate (ATP) synthase have been suggested as binding factors, no reliable CHIKV receptor has yet been discovered in mosquito cells [28,29]. The binding of the virus to cell receptors is followed by endocytosis [30], membrane fusion caused by low pH, and the transport of the viral nucleocapsid to the cytoplasm [31]. The replication cycle is completed in just about 4 h, which is exceptionally fast.

It is thought that CHIKV infection, as with other infections by arboviruses, produces lifelong immunity. In this regard, CHIKV-specific neutralizing antibodies have been detected 2 weeks after infection, and anti-CHIKV IgG antibodies persist for years [11,32].

## 3. Cell Metabolic Reprogramming 

In addition to the innate immune signaling pathways triggered by a viral infection, metabolic changes take place, and the field of immunometabolism is blossoming [33,34,35]. There is growing recognition that cellular metabolism is critical for host defenses against various infectious diseases [3,36]. The six major metabolic pathways associated with metabolic reprogramming are glycolysis, tricarboxylic acid cycle (TCA), pentose phosphate pathway (PPP), fatty acid synthesis (FAS), fatty acid oxidation (FAO), and amino acid synthesis [33]. Metabolic reprogramming was first identified in cells exposed to lipopolysaccharide (LPS) [33,37,38,39]; further understanding of this process in innate immunity is still based on LPS-stimulated macrophages as a model system [40,41,42,43,44]. However, metabolic changes in response to virus infection are increasingly appreciated [45,46,47,48,49]. 

Most of the energy required for non-dividing cells is generated through the TCA and glycolysis. Despite the efficiency of these catabolic processes, they do not automatically promote the synthesis of nucleic acids, amino acids, and lipids, which are equally necessary for the viral replicative process. Thus, viruses need to actively redirect some of the TCA cycle’s metabolic intermediates in the direction of anabolic pathways. To achieve this, viruses make use of complex mechanisms that allow them to regulate host cell metabolic pathways [50].

## 4. Viral Infection-Related Cell Metabolism

**Figure 1:**
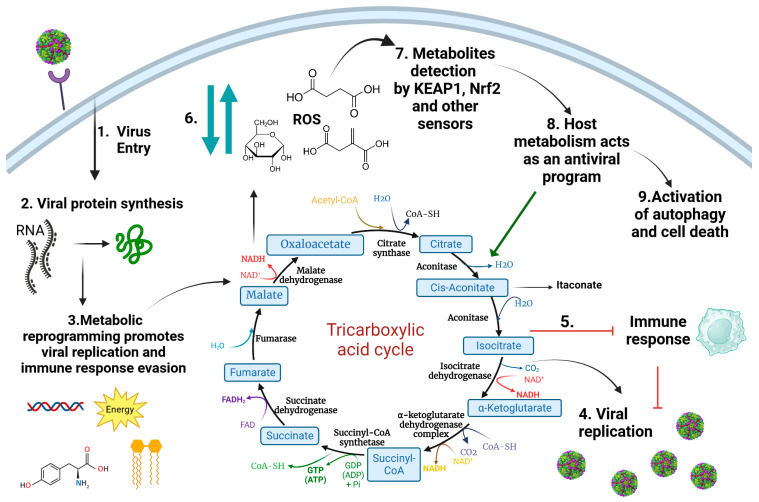
Metabolic reprogramming induced after viral infection. Upon the entry of viruses (1), several viral proteins are synthetized (2), changing the central metabolism of infected cells (3). Those changes in metabolism ensure energy production and the synthesis of the biomolecules required to build viral progenitor particles (4), and could also be involved in immune response evasion mechanisms (5). Changes in central metabolism inevitably lead to alterations in the abundance of different cellular metabolites, such as itaconate, glucose, lactate, succinate, and by-products such as ROS (6). Metabolite abundance is detected by host cell sensors, which include the KEAP1/Nrf2 complex (7). These metabolite sensors induce the expression of factors targeting the host metabolism (8) and activate other pathways (9) leading to the blocking of viral replication. Abbreviations: ROS: reactive oxygen species; KEAP1/Nrf2: Kelch-like ECH-associated protein 1/erythroid nuclear factor-related factor 2.

The TCA cycle is at the center of viral infection, replication, and pathogenesis, as TCA intermediates play important roles in inflammatory/anti-inflammatory homeostasis (Figure 1). For instance, lipids are necessary for the final assembly and budding phases in enveloped viruses, and the TCA cycle supplies the precursor (citrate) for fatty acid synthesis. In addition, itaconate, a cis-aconitate derivative of the TCA cycle, is a signaling molecule with anti-inflammatory, antioxidant, and antimicrobial properties [51]. 

Viral infections could induce changes in the concentration of various metabolites, and metabolomics approaches have been proposed for the discovery of biomarkers as a way to provide better diagnoses and clinical treatments [52,53]. The global metabolome of CHIKV-infected patient sera has identified the TCA cycle as a relevant metabolic pathway at play in the course of infection [54]. In addition, metabolic intermediates might provide a way for the host to detect a viral infection and then initiate antiviral programs [35]. The host metabolism itself is a target for several antiviral programs. This includes antiviral interferons (IFNs), which directly target or inhibit certain IFN-stimulated genes to deplete host metabolites needed for the virus [55].

On the other hand, recent research has demonstrated a connection between the immune system and the circadian rhythmicity in the context of viral infections [56]; it has been proposed that for a better clinical management of viral infections, such as for SARS-CoV-2, the circadian clock should be taken into account [57]. The interplay between viral infection and metabolism has also been analyzed [45,46,47,48,49,50,58], but less so the circadian/metabolic axis in the viral infection context; hence, circadian immunovirometabolism is an emerging area of interest to be considered for future studies on immunometabolism. 

## 5. Circadian Rhythmicity

To synchronize the internal physiological activities with the external time of day, most living organisms evolved a circadian system. Circadian rhythms are intrinsic oscillations that drive the cycling of an organism’s biological processes over a 24 h period. The circadian system affects a broad range of biological processes, such as feeding behavior, body temperature, metabolism, and immune response [59,60].

Circadian rhythms are governed by a “master clock” that resides in specialized cells of the mammalian suprachiasmatic nucleus (SCN), located in the anterior region of the hypothalamus. The transmission of light from the outside environment to the SCN via melanopsin photosensitive ganglion cells of the retina occurs through a network of interconnected positive and negative feedback loops enabling the SCN’s neurons to function with a 24 h rhythmicity. The loops that drive the 24 h rhythms constitute the molecular clock. Importantly, the cell clock machinery may be found in almost every type of cell, including immune system cells [61].

At least 10% of all transcripts are circadian-controlled in most tissues, demonstrating the molecular clock’s significant influence over the genome [61]. Circadian clock manifestations result from the circadian genes’ cyclic expression, which is regulated by E-box elements in the promoters and is activated by the complex of the transcriptional activators CLOCK (Locomotor Output Kaput) and BMAL1 (Brain Muscle Arnt-Like Protein 1). The period (PER) and cryptochrome (CRY) proteins (PER1, PER2, PER3 and CRY1, CRY2), as well as CK1 (also expressed by circadian genes), are CLOCK-BMAL1 repressors [62,63,64,65,66]. In addition to this main transcription–translation feedback loop, the regulators REV-ERB (nuclear receptor subfamily 1, group D, member 1) and RORα (retinoid-related orphan receptor α) bind to the ROR response elements in the Bmal1 promoter to activate or inhibit its transcription, respectively. This secondary "loop" further stabilizes rhythmicity [65]. 

The BMAL1-CLOCK heterodimer can potentially bind to thousands of promoters throughout the genome, and the oscillation of these binding causes the circadian expression of clock-controlled genes. Some of the cellular processes found to be clock-regulated include protein and epigenetic modifications, subcellular localization, translation rates, oxidation-reduction (redox) reactions, and ion concentrations [61].

Targeting circadian rhythms for disease prevention and therapy, including antiviral therapy, is becoming a feasible approach due to the better understanding of the molecular and cellular mechanisms underlying circadian physiology and pathophysiology [57,58], as well as the limitations of such an approach.

## 6. The Circadian Cycle, Metabolic Reprogramming, Immune Response, and Viral Infection Connection

**Figure 2:**
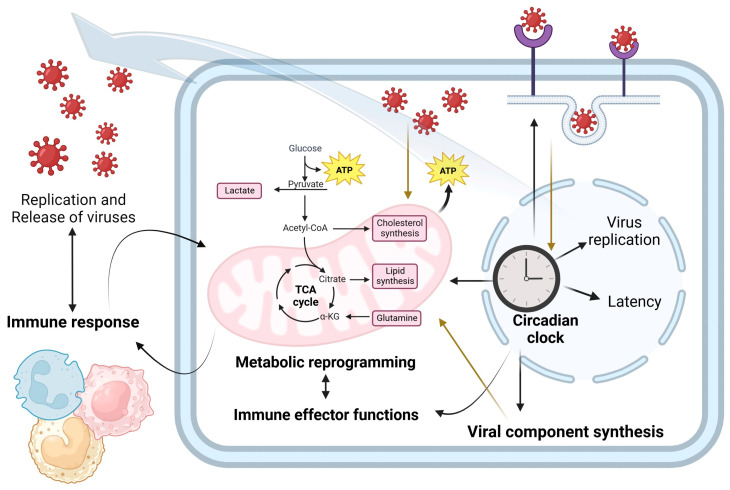
Circadian immunovirometabolism: connections between the cell clock, metabolic reprogramming, and the immune response in the context of viral infection. The cell clock has an important role in viral replication, at multiple stages of the viral replication cycle including the regulation of entry receptors and the anabolic pathways required for viral particle synthesis. In addition, infection can disrupt circadian rhythmicity and viruses might induce metabolic changes in infected cells to favor their replication while avoiding immune antiviral responses.

Circadian rhythms influence metabolic pathways [67], the immune response is endowed with circadian rhythmicity [7,8,68,69,70], and the immune system capacity to control an ongoing viral infection is time-of-day-dependent [5,6,56]. Thus, studies connecting those four elements are paving the way for a brand-new field of research: circadian immunovirometabolism (Figure 2). 

The circadian clock regulates, by means of the heterodimer CLOCK-BMAL1, a wide range of metabolic pathways, directly through the transcriptional activation of genes encoding key metabolic enzymes or indirectly through the control of factors that regulate tissue metabolisms [59,60]. For instance, BMAL1 inhibits the transcription of the enzyme pyruvate kinase M2 (PKM2), which converts phosphoenolpyruvate into pyruvate during glycolysis [71]. In addition, glucose absorption, serum levels of cholesterol, triglycerides, apolipoproteins, insulin, glucagon, leptin, and cortisone, as well as the amount of glucose transporters, glucagon receptors, and enzymes related to glycolysis, are also time-of-day-dependent [72].

The disruption of the circadian clock results in inflammation. Monocytes and macrophages lacking BMAL1 produce higher amounts of pro-inflammatory cytokines and chemokines and lower levels of the anti-inflammatory cytokine interleukin (IL)-10 [8,73,74,75,76]. The response to LPS-induced endotoxemia in humans is different depending on whether exposure to LPS occurs during the day or at night. Subjects injected with low doses of LPS at midday show higher levels of IL-10, whereas exposure to LPS after midnight increases the levels of tumor necrosis factor (TNF) and IL-6 [8,77]. The circulating levels of neutrophils and lymphocytes appear to be highest between 16:00 and 24:00 hours and lowest between 04:00 and 12:00 hours [4,78]. 

The redox balance is essential for both the circadian rhythmicity and the immune system cells’ function [79]; in this regard, the BMAL1-CLOCK heterodimer regulates the antioxidant defense in the lungs, through the rhythmic transcriptional activation of the antioxidant transcription factor Nfr2 [80]. 

Circadian cycles enable a more effective use of energy resources, allowing the immune system to prepare for pathogen exposure. The time of day and BMAL1 expression have been linked to the quality of the immune response against pathogens and their byproducts [81,82]. Likewise, time-of-day variation in response to bacteria is lost, and LPS-induced mortality increases when BMAL1 is deleted in myeloid cells [74,83]. CLOCK, BMAL1, and REV-ERB regulate the expression of the pattern recognition receptors (PRRs) that recognize viral nucleic acids during viral infections, and cells lacking Bmal-1 are more vulnerable to infection by RNA virus infection [9].

Majumdar et al. have highlighted the function of BMAL1 as a regulator of innate immunity [9]. In a viral model of acute and chronic airway disease, the deletion of the Bmal-1 gene or the environmental disruption of circadian rhythmicity exacerbated acute bronchiolitis caused by the Sendai virus (SeV) and influenza A virus in mice [84]. There is evidence that the SARS-CoV-2 virus’ entry into the viral replication cycle is affected by the circadian clock machinery, in particular by BMAL1 [85]. 

Flaviviruses also exhibit a relationship with the molecular components of the circadian clock. For instance, the hepatitis C virus (HCV) replication cycle includes numerous stages that are influenced by BMAL1 and REV-ERB, including particle entrance into hepatocytes and RNA genome replication. By interfering with lipid signaling pathways, the genetic deletion of Bmal1 and the overexpression or activation of REV-ERB with synthetic agonists prevent the replication of HCV and the related flaviviruses, dengue and Zika [86]. Table 1 shows some examples of the relationship between circadian clock components and viral infection. 

## 7. Phagocytic Cells’ Response against CHIKV and Its Role in Viral Pathogenesis

Throughout the course of infection, CHIKV targets monocytes and macrophages, among other cells, and high viremia is the result of viral replication in the liver and spleen [13]. Infected endothelial cells, monocytes, and macrophages have been considered to be “Trojan horses”, since they help in viral spreading to reservoir areas of the body [90,91]. It has recently been shown that human synovial tissues are CHIKV sanctuaries since this RNA virus is still detectable in joint fluids up to 18 months after infection [92].

Monocytes and macrophages provide protection against infections, as components of the innate immune system. These cells express different pattern recognition receptors (PRRs) such as Toll-like receptors (TLRs), nucleotide oligomerization domain (NOD)-like receptors, and retinoic acid inducible gene-I (RIG-I)-like receptors (RLRs), all of which detect conserved structures in viruses, referred to as pathogen-associated molecular patterns (PAMPs); in turn, these PRRs induce intracellular signaling cascades that lead to the activation of transcription factors, and the subsequent transcription of genes encoding pro-inflammatory cytokines, chemokines, and type I interferons, whose function is to induce inflammation, regulate the immune response, and limit viral replication [93]. In spite of this immune regulation of CHIKV, a protracted polyarthritis akin to chronic inflammatory rheumatism may occur [94], where the infection of satellite cells and myoblasts connected to skeletal muscle, as well as of myofibers, takes place [95,96], resulting in the necrosis of muscle tissue and myalgia [97,98]. 

Monocytes and monocyte-derived macrophages appear to play a central role in CHIKV-associated joint pathology, as the infection of these cells leads to the production of pro-inflammatory cytokines (TNF-α and IL-6), the influx of macrophages, T and B lymphocytes, and natural cytolytic lymphocytes, all of which contribute to the inflammatory environment and tissue damage associated with arthritic pain. In spite of powerful interferon-mediated and humoral immune responses that help systemic virus clearance to undetectable levels in the peripheral blood within days, the long-term infection of synovial macrophages is thought to be a significant component of sustained immune reactivity in joint tissue, contributing to chronic arthritic pain [99].

CHIKV infection in non-human primates resembles the viral, clinical, and pathological characteristics seen in CHIKV-infected human patients. The long-lasting CHIKV disease symptoms seen in humans could be explained by the long-term CHIKV infection shown in the macaques’ joints, muscles, lymphoid organs, and liver [90,91]. During the advanced phases of CHIKV infection in vivo, macrophages serve as the primary cellular reservoirs [91]. Interestingly, only a portion of human monocytes/macrophages are infected in vitro, resembling the brief innate immune response in mice that rapidly subsides after 48 h of infection [100].

Nevertheless, understanding the role of phagocytic cells, and in particular the susceptibility of monocytes and macrophages to CHIKV infection and their ability to control CHIKV persistence, is still at an early stage.

## 8. The Tricarboxylic Acid (TCA) Cycle as the Immunovirometabolic Center in Macrophages

Infecting viruses are likely to alter the function of macrophages in such a way as to limit their anti-viral mechanisms, allowing viruses to persist in a dormant state until a more appropriate time for viral reactivation [101].

The TCA cycle constitutes one of the most important hubs for the connection between cell metabolism and antiviral immune response in macrophages [51], and it is also a central regulator for directing intracellular metabolic adaptation, intracellular signaling, and pro-inflammatory and anti-inflammatory responses [102]. 

During classical macrophage activation, the TCA cycle intermediates succinate and citrate accumulate, and these metabolites harbor signaling properties that influence inflammatory gene expressions. Macrophage polarization towards the M1 or M2 phenotypes is dependent on glycolysis and oxidative phosphorylation, respectively [39,103,104,105]. 

On the other hand, the electron transport chain, linked to the TCA cycle through succinate dehydrogenase (complex II), is also altered during infections, generating reactive oxygen species by complexes I and III [106]. Alternatively, activated macrophages undergo epigenetic reprogramming that is dependent on a-ketoglutarate, another TCA intermediate, to induce the activation of anti-inflammatory genes [107]. 

The vaccinia virus (VACV) is one of the most studied examples of viral infections leading to the rewiring of the TCA cycle. The upregulation of TCA cycle intermediates, including citrate, has been described in VACV-infected cells, and VACV infection also increases oxidative phosphorylation, resulting in increased ATP synthesis [108].

The reprogramming of the TCA cycle has also been shown in human cytomegalovirus (HCMV) infection. For HCMV, the fundamental role of the TCA cycle is the production of ATP and NADH, which enable cells to carry out the energetically costly anabolic reactions required for the synthesis of viral proteins and nucleic acids and to envelop lipids. HCMV infection also increases glutamine catabolism to replenish the TCA cycle and support the energy supply [109].

The SARS CoV-2 virus is particularly important since the cells that contribute most to COVID-19 pathology are macrophages. Recent studies suggest that increased glycolysis in proinflammatory macrophages leads to the accumulation of the TCA cycle intermediates succinate, citrate, and itaconate, thus changing the expression of inflammatory genes. The accumulation of succinate has been highlighted since its oxidation in mitochondria by succinate dehydrogenase (mitochondrial respiratory complex II) initiates the reverse electron transport (RTE) that promotes the generation of ROS, indispensable for SARS-CoV-2 replication [110,111]. 

Cells infected with the hepatitis C virus (HCV) show increased concentrations of TCA cycle enzymes and several components of the electron transport chain. Furthermore, proteomic analyses have revealed an increase in pyruvate carboxylase, the mitochondrial enzyme that generates oxaloacetate [112].

The infection of human foreskin fibroblasts with ZIKV increases glucose uptake and its use in the TCA cycle and for amino acid synthesis. In contrast, in C6/36 mosquito cells, ZIKV infection increases glucose use in the pentose phosphate pathway, highlighting the differences in metabolic reprogramming depending on which type of cell is infected [113].

Virus-infected cells in which metabolic reprogramming has specifically taken place in the TCA cycle show alterations in the levels of some intermediary metabolites such as malate, fumarate, and itaconate, which are sensed through the NRF2/KEAP1 complex. This molecular mechanism is still under intensive research, and the most convincing results so far come from experiments using derivatives of itaconate (4-octyl-itaconate (OI) and fumarate (dimethyl fumarate, DMF), two compounds shown to inhibit the replication of the SARS-CoV-2 virus. 

Initial RNA-Seq-based experiments showed a downregulation of Nrf2-dependent genes in COVID-19 patients, indicating that a potential host evasion mechanism for SARS-CoV-2 could involve the impairment of the Nrf2 pathway. It has been demonstrated that SARS-CoV-2 replication, as well as the replication of other pathogenic viruses, including herpes simplex virus type 1 (HSV-1) and vaccinia, are all inhibited by the activation of Nrf2 by OI or DMF and the suppression of KEAP1; experimental evidence suggests that the anti-viral mechanism is independent of interferons [114]. An itaconate-mediated anti-inflammatory response, which is independent of NRF2, has also been described, indicating the existence of some other metabolic sensors [115]. The infection of neurons with the ZIKV induces IRG1, the enzyme that drives the synthesis of itaconate, by a complex Z-DNA binding protein (ZBP1)/receptor-interacting protein kinase 3 (RIPK3)/interferon regulatory factor 1(IRF1)-dependent mechanism, which, in turn restricts ZIKV replication. Itaconate inhibits the activity of succinate dehydrogenase and generates a metabolic state in neurons that suppresses the replication of viral genomes [3]. 

These aforementioned examples highlight the importance of metabolic reprogramming in the course of viral infections, specifically the central role played by the TCA cycle metabolic intermediates and derivatives such as succinate and fumarate, and itaconate, respectively, in inflammatory/anti-inflammatory responses, as well as in anti-viral mechanisms. Most studies on metabolic reprogramming have addressed DENV and ZIKV infections rather than CHIKV infection. However, since DENV and ZIKV share many characteristics with CHIKV, another arbovirus, from transmission mechanisms to the virus replication cell cycle, and considering the elicited immune response, it could be suggested that CHIKV also shares with DENV and ZIKV some metabolic reprogramming-based mechanisms for immune evasion. However, a note of caution should be added since, as has been mentioned in the previous sections, infection with the same virus may have different outcomes in different cell types. 

## 9. Conclusions and Perspectives

Chikungunya, Zika, and dengue induce different degrees of illness, with a high burden on public health and the economy of the countries in which they are endemic. To date, there has been no effective or specific treatment for these three viral diseases and most therapies are directed to relieving the symptoms. Studies aimed at understanding the metabolic reprogramming upon viral infection are underway and eventually this might help to develop new drugs and therapeutic approaches to deal with viral diseases.

These studies have shown how TCA cycle metabolic intermediates such as citrate and succinate can exert functions on the innate immune cells. Citrate accumulates in macrophages following the stimulation of Toll-like-receptor (TLR)-4, and promotes the synthesis of prostaglandins, nitric oxide (NO), and ROS [116], whereas succinate has been recognized as a signaling metabolite that induces the synthesis of IL-1β in a hypoxia inducible factor (HIF)-1α-dependent manner [117]; the antiviral role for itaconate has also been demonstrated [3]. 

The resurgence of CHIKV demands further research. Studies on the metabolic reprogramming of the immune cells susceptible to CHIKV infection, such as macrophages, could offer a suitable starting point. Moreover, since the communication between immunity, metabolism, and circadian rhythmicity has been widely recognized, the circadian rhythmicity variable should be included in future CHIKV-infection experimental designs. Expanding the circadian immunovirometabolism knowledge will allow the redirection of the immune response and the virus replicative cycle to a more effective treatment of viral diseases. Studies on the role of different metabolites and metabolic enzymes in the anti-CHIKV immune response and inflammatory processes might offer the opportunity to design molecules that target metabolism, with a therapeutic potential not only against CHIKV but also as an immunomodulator for other pathologies.

## Figures and Tables

**Table 1:** Cell clock components and viral infection. Abbreviations: respiratory syncytial virus (RSV), parainfluenza virus type 3 (PIV3), trans-activator of transcription (Tat), dengue virus (DENV), Zika virus (ZIKV), human immunodeficiency virus (HIV).

Virus	Clock Component	Effects	References
RSV, PIV3	BMAL1	Bmal1-deficient cells were more susceptible to these infections.	[9]
SeV Influenza A	BMAL1	Deletion of Bmal1 increased viral susceptibility and impaired control of viral replication.	[84]
SARS-CoV-2	BMAL1	Silencing of Bmal1 inhibits virus entry into the cell and reveals that the viral cycle is influenced by a circadian cycle.	[85]
HCV DENV ZIKV	BMAL1 and REV-ERBα	BMAL1 and REV-ERBα affect HCV entry into hepatocytes and genome replication of HCV, DENV, and ZIKV.	[86]
Herpesvirus	BMAL1 and CLOCK	The virus increases the production of BMAL1, and low levels of BMAL1 increase infection. CLOCK activates the expression of the entry receptor by attaching to its promoter.	[87,88]
HIV		The HIV Tat protein affects circadian rhythmicity.	[89]
